# Supplementary material for: Pyruvate Dehydrogenase Kinase 1 inhibition mediated oxidative phosphorylation enhancement in cartilage promotes osteoarthritis progression
Source: BMC Musculoskelet Disord. 2023 Jul 20;24:597. doi: 10.1186/s12891-023-06585-6 (PMC10357736; doi:10.1186/s12891-023-06585-6)
Supplement: Supplementary file 1 — Additional file 1. [file 12891_2023_6585_MOESM1_ESM.zip › MF.Gsea.1653627316242/GOMF_POLY_PYRIMIDINE_TRACT_BINDING.html]

Details for gene set GOMF\_POLY\_PYRIMIDINE\_TRACT\_BINDING[GSEA]

|  || Dataset | 55457\_collapsed\_to\_symbols.cls.cls #OA\_versus\_normal.cls.cls #OA\_versus\_normal\_repos |
| Phenotype | cls.cls#OA\_versus\_normal\_repos |
| Upregulated in class | normal |
| GeneSet | GOMF\_POLY\_PYRIMIDINE\_TRACT\_BINDING |
| Enrichment Score (ES) | -0.5524954 |
| Normalized Enrichment Score (NES) | -1.6731346 |
| Nominal p-value | 0.0052083335 |
| FDR q-value | 0.115934305 |
| FWER p-Value | 0.942 |
Table: GSEA Results Summary

  

Fig 1: Enrichment plot: GOMF\_POLY\_PYRIMIDINE\_TRACT\_BINDING      
 Profile of the Running ES Score & Positions of GeneSet Members on the Rank Ordered List

  

| SYMBOL | TITLE | RANK IN GENE LIST | RANK METRIC SCORE | RUNNING ES | CORE ENRICHMENT || 1 | ATXN1 | ataxin 1 [Source:HGNC Symbol;Acc:HGNC:10548] | 2862 | 0.264 | -0.1845 | No |
| 2 | KHDRBS1 | "KH RNA binding domain containing, signal transduction associated 1 [Source:HGNC Symbol;Acc:HGNC:18116]" | 3748 | 0.182 | -0.2273 | No |
| 3 | MSI1 | musashi RNA binding protein 1 [Source:HGNC Symbol;Acc:HGNC:7330] | 3893 | 0.165 | -0.2148 | No |
| 4 | MCRS1 | microspherule protein 1 [Source:HGNC Symbol;Acc:HGNC:6960] | 4286 | 0.129 | -0.2269 | No |
| 5 | KHDRBS2 | "KH RNA binding domain containing, signal transduction associated 2 [Source:HGNC Symbol;Acc:HGNC:18114]" | 4550 | 0.109 | -0.2317 | No |
| 6 | PTBP1 | polypyrimidine tract binding protein 1 [Source:HGNC Symbol;Acc:HGNC:9583] | 4650 | 0.100 | -0.2251 | No |
| 7 | RBMS1 | RNA binding motif single stranded interacting protein 1 [Source:HGNC Symbol;Acc:HGNC:9907] | 7121 | -0.090 | -0.4040 | No |
| 8 | HNRNPC | heterogeneous nuclear ribonucleoprotein C [Source:HGNC Symbol;Acc:HGNC:5035] | 7437 | -0.114 | -0.4122 | No |
| 9 | FMR1 | FMRP translational regulator 1 [Source:HGNC Symbol;Acc:HGNC:3775] | 7944 | -0.151 | -0.4300 | No |
| 10 | RBMS2 | RNA binding motif single stranded interacting protein 2 [Source:HGNC Symbol;Acc:HGNC:9909] | 8373 | -0.184 | -0.4370 | No |
| 11 | PABPC4 | poly(A) binding protein cytoplasmic 4 [Source:HGNC Symbol;Acc:HGNC:8557] | 8741 | -0.212 | -0.4352 | No |
| 12 | RBMS3 | RNA binding motif single stranded interacting protein 3 [Source:HGNC Symbol;Acc:HGNC:13427] | 8981 | -0.230 | -0.4209 | No |
| 13 | HNRNPU | heterogeneous nuclear ribonucleoprotein U [Source:HGNC Symbol;Acc:HGNC:5048] | 9847 | -0.306 | -0.4444 | No |
| 14 | IFIT5 | interferon induced protein with tetratricopeptide repeats 5 [Source:HGNC Symbol;Acc:HGNC:13328] | 11240 | -0.462 | -0.4865 | Yes |
| 15 | SSB | small RNA binding exonuclease protection factor La [Source:HGNC Symbol;Acc:HGNC:11316] | 11955 | -0.584 | -0.4585 | Yes |
| 16 | U2AF2 | U2 small nuclear RNA auxiliary factor 2 [Source:HGNC Symbol;Acc:HGNC:23156] | 12226 | -0.652 | -0.3864 | Yes |
| 17 | PABPC3 | poly(A) binding protein cytoplasmic 3 [Source:HGNC Symbol;Acc:HGNC:8556] | 12257 | -0.663 | -0.2940 | Yes |
| 18 | HNRNPH1 | heterogeneous nuclear ribonucleoprotein H1 [Source:HGNC Symbol;Acc:HGNC:5041] | 12554 | -0.776 | -0.2063 | Yes |
| 19 | PABPC1 | poly(A) binding protein cytoplasmic 1 [Source:HGNC Symbol;Acc:HGNC:8554] | 12562 | -0.778 | -0.0957 | Yes |
| 20 | DAZAP1 | DAZ associated protein 1 [Source:HGNC Symbol;Acc:HGNC:2683] | 12686 | -0.854 | 0.0166 | Yes |
Table: GSEA details [plain text format]

  

Fig 2: GOMF\_POLY\_PYRIMIDINE\_TRACT\_BINDING      
 Blue-Pink O' Gram in the Space of the Analyzed GeneSet

  

Fig 3: GOMF\_POLY\_PYRIMIDINE\_TRACT\_BINDING: Random ES distribution      
 Gene set null distribution of ES for **GOMF\_POLY\_PYRIMIDINE\_TRACT\_BINDING**
